# The DNA Damage Response: A Common Pathway in the Regulation of NKG2D and DNAM-1 Ligand Expression in Normal, Infected, and Cancer Cells

**DOI:** 10.3389/fimmu.2013.00508

**Published:** 2014-01-07

**Authors:** Cristina Cerboni, Cinzia Fionda, Alessandra Soriani, Alessandra Zingoni, Margherita Doria, Marco Cippitelli, Angela Santoni

**Affiliations:** ^1^Department of Molecular Medicine, Istituto Pasteur-Fondazione Cenci Bolognetti, “Sapienza” University of Rome, Rome, Italy; ^2^Laboratory of Immunoinfectivology, Bambino Gesù Children’s Hospital, IRCCS, Rome, Italy; ^3^Mediterranean Neurological Institute, Pozzilli, Italy

**Keywords:** NK cells, stress, DNA damage response, NKG2D ligands, DNAM-1 ligands

## Abstract

NKG2D and DNAM-1 are two activating receptors, present on the surface of NK cells and other cells of the immune system. Their ligands – MICA, MICB, ULBP1-6 for NKG2D, PVR/CD155 and Nectin-2/CD112 for DNAM-1 – can be constitutively expressed at low levels in some normal cells, but they are more often defined as “stress-induced,” since different stimuli can positively regulate their expression. In this review, we describe the molecular mechanisms involved in the up-regulation of NKG2D and DNAM-1 ligands under different physiological and pathological “stress” conditions, including mitosis, viral infections, and cancer. We will focus on the DNA damage response, as recent advances in the field have uncovered its important role as a common signaling pathway in the regulation of both NKG2D and DNAM-1 ligand expression in response to very diverse conditions and stimuli.

## Introduction

The immune system is tasked with protecting the organism from pathogen attack, but also with patrolling cells and tissues that have been dysregulated by non-microbial challenges, such as ultraviolet radiation, heat shock, oxidative stress, or tumor transformation. From a certain point of view, all these responses are not completely unrelated. In fact, one prominent consequence common to different types of stressors is the up-regulation of the MHC class I-like proteins MICA, MICB, ULBP1-6, which are present at low to undetectable levels in normal cells, but can be induced both by infectious agents and by sterile stresses, including cell division and/or tumor transformation (1–4). These molecules are the ligands of the activating receptor NKG2D, a member of the C-type lectin-like superfamily of innate receptors, able – alone or in combination with other receptors – to activate the effector functions of NK cells, CD8^+^ T cells, γδ T cells, and a subset of CD4^+^ T cells (1). Though less characterized, DNAM-1 is another activating receptor expressed by cytotoxic lymphocytes, and its ligands PVR and Nectin-2, two adhesion molecules belonging to the Ig-like superfamily, are similarly induced by cellular stresses (5–9). Thus, expression of ligands for activating NK cell receptors appears to be a critical mechanism of immunosurveillance against stressed cells (10). In addition, recent studies demonstrated that another shared aspect of stress responses consists in the activation of the DNA damage response (DDR), a major signaling pathway implicated in the up-regulation of ligand expression (11).

DNA must be protected from damage produced spontaneously during DNA replication or from endogenously generated reactive oxygen species (ROS) that are a byproduct of normal metabolic processes. In addition, a plethora of external stimuli, such as ultraviolet light, ionizing radiation, and viral infections can cause DNA lesions (both in a ROS-dependent and -independent manner) that can block genome replication and transcription (12). Therefore, the general term DDR is related to a complex series of cellular stress-induced pathways that detect DNA damage and that are involved in the maintenance of genome integrity and avoidance of mutated DNA duplication (13). Three members of the phosphatidylinositol 3-kinase-like serine/threonine protein family are central to this response: ATM, ATR, and the DNA-dependent protein kinase (DNA-PK) (14, 15). Both ATM and DNA-PK are known to be recruited to and activated by double-stranded DNA breaks, while ATR is activated by stalled replication forks and subsequently single-stranded DNA breaks (16–18). Following the recognition of DNA lesions by sensor proteins, these kinases activate many downstream mediators, such as the serine/threonine kinases Chk1 and Chk2, able to phosphorylate many effector proteins that induce either cell-cycle arrest and DNA repair or, if unsuccessful, initiation of programs instructing the cell to undergo apoptosis or enter terminal differentiation through senescence (12–14).

## Normal Cells

There is a substantial body of evidence showing the involvement of DDR in many physiological processes, such as mitosis (19), insulin response (20), V(D)J recombination (21, 22), or after lipopolysaccharide stimulation in macrophages (23). In addition, the self-renewal capacity of hematopoietic stem cells was found to depend on an ATM-mediated modulation of the response to oxidative stress (24). Enhanced phosphorylation of either ATM or one of its substrates, the histone H2AX, as well as the increase of ATM protein levels were observed on T cells upon activation in response to a plethora of stimuli (8, 25–27).

**Figure 1:**
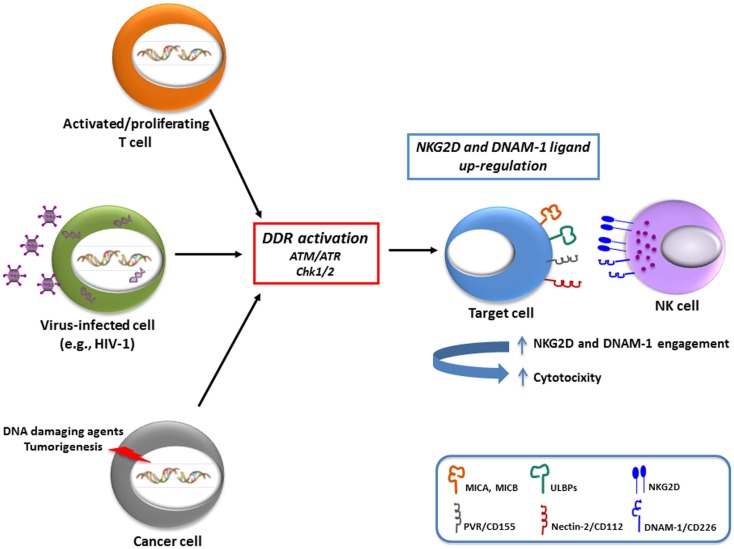
**Schematic representation of the variety of stimuli that can up-regulate NKG2D and DNAM-1 ligands**. There is evidence that both in normal cells (e.g., antigen-activated T lymphocytes), as well as in pathological conditions, including virally-infected cells (in particular with HIV-1) and cancer cells, a major regulatory pathway involved in ligand up-regulation is the DNA damage response (DDR), activated by different stimuli. The increased expression of activating ligands has been shown to be implicated in the recognition and elimination of “stressed” cells by NK cells, and presumably also by other cytotoxic cells (i.e., γδ T cells and CD8^+^ T cells).

In relation to activating ligands, studies performed in our own and other laboratories have shown that MIC, ULBP, and PVR molecules are induced on antigen-activated T cells (8, 27–29) (Table 1). Interestingly, both oxidative stress (mainly mediated by a macrophage-dependent production of ROS) and DDR were implicated in the induction of MICA and PVR on activated T cells (8, 27), suggesting that signaling via ATM/ATR kinases and DDR could represent a common pathway regulating the expression of NKG2D and DNAM-1 ligands on T lymphocytes (Figure 1). Of note, PVR and NKG2D ligand expression on T cells was mainly associated with progression to the S and G_2_/M phases of the cell (8) (and our unpublished observations). Since ATM/ATR are known to be regulators of cell division, the increased cellular proliferation upon antigenic stimulation could be the crucial signal resulting in NKG2D and DNAM-1 ligand expression on healthy cells. In fact, a correlation of either NKG2D ligand or PVR expression with cell proliferation has been documented in several studies. Expression of MICA has been shown in fast dividing tissues including the gut epithelium (30) and in highly proliferating cell lines (31). Indeed, high surface MICA expression was detected in fibroblasts during the stage of rapid growth and was strongly down-regulated following cell–cell contact (32). Similarly, PVR expression in epithelial cells was tightly regulated by changes in cell density (33). Groh and colleagues have also demonstrated that the presence of MIC molecules on rheumatoid arthritis synoviocytes was strongly associated with the expression of the nuclear Ki-67 proliferation marker (31). A recent study indicated that MICA expression levels on endothelial cells were substantially increased by the induction of cell proliferation mediated by FGF-2 or wound healing (34). These *in vitro* observations were further supported by *in vivo* studies performed in rodents. Using bromodeoxyuridine incorporation in murine bone marrow grafts, RAE-1 (the murine orthologs of ULBP proteins) was detected on a large fraction of donor proliferating progenitor cells in the spleen of the transplant recipients rather than on the long-term hematopoietic stem cells (35), and in relation to DNAM-1 ligands, a study in the rat showed that the presence of PVR in the liver was confined to proliferating hepatocytes during liver regeneration (33). When the transcriptional regulation of activating ligands was analyzed in normal proliferating cells, their expression was reported to depend on NF-kB, Sp1, and the E2F family of transcription factors (27, 36–38).

**Table 1:** **DDR-dependent up-regulation. of NKG2D and DNAM-1 ligand expression**.

Activator	Cell model	Activating ligand	Reference
ATM, ATR	T lymphocytes	MICA	(27)
ATM, ATR	T lymphocytes	PVR	(8)
ATM	LPS-stimulated macrophages	MICA	(23)
ATR	HIV-1 infected T cells	NKG2D ligands	(46, 47)
ATR	HIV-1 infected T cells	PVR	(50)
ATM, ATR, Chk1	Hepatoma	MICA/B	(69)
ATM	Hepatoma	MICB	(68)
ATM, ATR	Cervical and colon carcinoma, T cell leukemia	ULBP2	(67)
ATM, Chk2	Multiple myeloma	MICA	(65)
ATM/ATR	Multiple myeloma	MICA/B, ULBP1-3	(7)
ATM/ATR	Ewing sarcoma	MICB	(70)
ATM, Chk2	Colon cancer cells	MICA/B, ULBP1-3	(71)
p53	Lung cancer	ULBP1-2	(73)
p53	Colon/breast cancer	ULBP2	(74)
ATM, Chk1	Murine ovarian tumor cells	RAE-1	(11)
ATM	Murine B cell leukemia	PVR	(66)

The biological significance of an increased expression of both NKG2D and DNAM-1 ligands on the surface of dividing cells could be to alert the immune system of a potentially dangerous cell-cycle progression. Indeed, Davis’s group reported that human NK cells bound to cells in mitosis more efficiently than the same cells in other stages of the cell cycle (39) and our studies further demonstrated that proliferating T cells become more susceptible to NK cell-mediated recognition and killing (8) (Figure 1). Thus, NK cell restriction of T cell responses might be relevant in the maintenance of lymphocyte homeostasis as well as in the context of autoimmunity or graft-versus-host disease (3).

## Virally-Infected Cells

Infection by several viruses, including herpesviruses, adenoviruses, papillomaviruses, and retroviruses, is sufficient to activate some or all of the DDR-mediated repair pathways. Simplistically, this was perceived as recognition by the host cell of the incoming genetic material as its own damaged DNA, but it is now considered to be, at least in part, an anti-viral response aimed at combating the pathogen by posing a threat to viral genome integrity and replication (40). However, viruses have evolved a complex relationship with the DDR pathway being able to either inhibit or exploit DDR components in order to favor their own replication process, with some viruses using both strategies in a spatially and temporally orchestrated manner (41, 42). From a theoretical point of view, the viral-induced activation of DDR and the consequent up-regulation of the ligands for activating receptors could render infected cells susceptible to the recognition and elimination by cytotoxic lymphocytes, thus contributing to the anti-viral response. In humans, up-regulation of NKG2D and/or DNAM-1 ligands was indeed observed following infection by several viruses (e.g., HCMV, HCV, EBV, HIV-1) (43, 44), but the link between this phenomenon and DDR activation has been investigated only for HIV-1. Studies performed in our own and other laboratories have shown that HIV-1 infection of CD4^+^ T lymphocytes up-regulates both MIC and ULBP proteins, especially ULBP2, as well as PVR, and thus exposes infected cells to recognition and lysis by NK cells (45–49) (Figure 1). Recently, the HIV-1 Vpr protein was identified as the key viral factor responsible for the up-regulation of both NKG2D ligands and PVR in infected CD4^+^ T cells (46, 47, 50) (Table 1). The stimulatory effect of Vpr on ligand expression relies on its capacity to recruit a cullin-ring E3 ubiquitin ligase (DDB1-CUL4A) and to activate ATR (46, 51). The same Vpr interactions ultimately lead infected cells to arrest in G_2_, a phase of the cell cycle that allows efficient virus production (52, 53), therefore it is possible that ligand up-regulation is secondary to G_2_ arrest. Apparently, the effects of Vpr on ULBP2 and PVR expression operate at different levels, since ULBP2 but not PVR transcripts accumulate in Vpr-expressing cells (46, 47, 50). Thus, additional work is clearly needed to understand how Vpr up-regulates each ligand.

As a countermeasure for ligand up-regulation, HIV-1 as well as many other viruses, have developed the capacity to inhibit cell-surface ligand expression. For HIV-1, this activity is mediated by the viral proteins Nef, Vif, and Vpu that down-regulate NKG2D ligands and/or PVR, and, as a consequence, decrease the susceptibility of HIV-infected cells to NK-cell-mediated lysis (45, 48, 54). Interestingly, T cells infected with a mutated virus defective for the expression of the two proteins, Vpr and Nef, that exert opposite effects on NKG2D ligand and PVR expression, display higher ligand levels compared to uninfected cells (50) (and our unpublished data), suggesting the existence of an additional Vpr-independent mechanism of ligand up-regulation. This mechanism may be related to the previously reported triggering of ATM during HIV-1 DNA integration (55).

In sum, a picture is emerging in which HIV-1 hijacked some cellular DDR effector molecules that are required for efficient viral replication and, at the same time, has developed means to contrast the effect of DDR activation on NKG2D and DNAM-1 ligand expression that is dangerous for the virus itself. The fact that also several other viruses (e.g., HCMV, KSHV, HCV, HAdV, HHV, HCV) have evolved the capacity to down-regulate NKG2D and DNAM-1 ligands, suggests that activating NK cell receptors and host immune responses mediated by NKG2D and DNAM-1 represent a serious threat that a virus must circumvent. Interestingly, these viruses are known to interact at some point of their life cycle with at least one component of the DDR machinery to aid their own replication. Therefore, a better understanding of the dual (pro- and anti-viral) role of DDR in the life cycle of HIV-1 and of other viruses may lead to new strategies aimed at suppressing viral replication while maintaining and, possibly, reinforcing anti-viral immune responses.

## Cancer Cells

The relevance of NKG2D in tumor surveillance has been demonstrated by *in vivo* experiments showing that overexpression of NKG2D ligands in cancer cells causes tumor rejection in mice (56, 57), and that NKG2D-deficient animals are defective in tumor surveillance in models of spontaneous malignancy (58). In humans, it has been shown that many tumors up-regulate NKG2D ligands, probably as a result of the oncogenic process itself, and this renders them more sensitive to recognition by NK and cytotoxic T cells (59–61). In relation to DNAM-1, *in vitro* studies have shown that this activating receptor triggers NK cell-mediated killing of a range of tumor cells expressing PVR and/or Nectin-2. Moreover, DNAM-1-deficient mice show an impaired clearance of PVR-expressing tumor cells and develop more tumors in response to chemical carcinogens (62).

In cancer cells, stress signals, and in particular those associated with DDR, induce both NKG2D and DNAM-1 ligand expression (7, 11) (Table 1). In fact, cells exposed to chemotherapeutic agents, genotoxic stimuli, or stalled DNA replication cycles, up-regulate NKG2D ligands through the activation of the DDR, suggesting that ATM, ATR, and Chk1 may be predominantly responsible for NKG2D ligand expression maintenance (11). These findings provided for the first time a link between the constitutive activation of DDR in tumors and the frequent up-regulation of NKG2D ligands in transformed cells, suggesting that constitutive ligand expression could be maintained by persistent genotoxic stress in tumor cell lines. Moreover, many evidences support the idea that DDR can be frequently activated in early neoplastic lesions, and probably NKG2D and DNAM-1 ligand induction by DNA damage represents a tumor surveillance mechanism operating at the very early stages of tumorigenesis, possibly increasing the sensitivity of damaged cells to NK- and/or T cell-mediated lysis (11, 63–66) (Figure 1). In particular, Croxford and colleagues have very recently provided the first *in vivo* evidence that T and NK cells play a critical role in the regression of B cell lymphomas in Eμ-myc mice, by showing that spontaneous rejection requires the expression of PVR on tumor cells, which is regulated by an ATM-initiated DDR (66). Studies from our laboratory have also contributed to better delineate the link between activation of DDR and regulation of NKG2D and DNAM-1 ligands. In particular, we demonstrated that genotoxic drugs, when used at doses that do not affect cell viability, induce the up-regulation of NKG2D and DNAM-1 ligand expression on several multiple myeloma cell lines and primary malignant plasma cells, and consequently enhance NK cell degranulation toward drug-treated tumor cells. This effect is dependent on the activity of ATM/ATR kinases, and occurs in combination with the establishment of a chemotherapy-induced senescent phenotype (7). Our observations in multiple myeloma are consistent with a number of other studies describing NKG2D ligand regulation by several DNA-damaging conditions (11, 65, 67–70) (Table 1). Moreover, Leung and colleagues have recently reported the up-regulation of NKG2D ligands by the aldosterone antagonist spironolactone through the DNA damage-independent activation of ATM-Chk2 in multiple colorectal cancer cells. The drug-mediated effect requires the activation of retinoid X receptor γ (RXRγ), probably capable of initiating chromatin remodeling, and results in activation of the ATM-Chk2 DNA repair checkpoint pathway that enhances NKG2D ligand expression (71). These observations demonstrate a key role for the protein kinases mediating DDR activation in the promotion of NKG2D ligand expression, and suggest that DNA lesions are not a prerequisite necessary to these effects.

One of the most extensively studied component of DDR is the tumor suppressor protein p53, and DNA damage leads to enhanced stability and activity of p53 upon its ATM-mediated phosphorylation (72). Conflicting results have been reported about the involvement of p53 in the regulation of NKG2D ligands, with data showing positive, negative, or no effect. Gasser and colleagues ruled out p53 from the mechanisms at the basis of genotoxic drug-induced NKG2D ligand up-regulation in mice and human cell lines, since the lack of p53 had no effect on NKG2D ligand expression after genotoxic stress (11). By contrast, other studies showed that ULBP1 and ULBP2 are direct p53 target genes in human cell lines and, accordingly, treatment of certain cancer cells with RITA, a small molecular compound that reactivates wild-type p53, resulted in the up-regulation of ULBP2 expression (73, 74). On the contrary, it has been recently shown that ULBP2 gene can be repressed via the p53-mediated increase in cellular miR-34 levels (75). Thus, the outcome of p53 activation on ULBP2, and possibly other NKG2D ligands, might depend on the context of its activation, pointing to a complex role of p53 that awaits further investigation.

A novel perspective in the regulation of MICA expression has been recently demonstrated by a study showing that up-regulation of MICA by genotoxic stress was enhanced by inhibiting STAT3 activity in both cancer and non-malignant cells (76). In agreement with this observation, studies conducted by our group have demonstrated that inhibition of STAT3 – obtained by using GSK3 kinase activity inhibitors – can enhance the expression of MICA induced by the chemotherapeutic drug melphalan in multiple myeloma cells (77). Therefore, these results add an additional layer of complexity in the molecular mechanisms regulating the expression of MICA and likely of other NK cell activating ligands.

## Conclusion

As shown in less of 10 years of intense research, NK cell activating receptors and their ligands represent an important warning system alerting cytotoxic lymphocytes of danger and stress signals. Since the expression of NKG2D and DNAM-1 ligands is rarely seen in normal cells, this means that small changes in their cell-surface levels may significantly influence the susceptibility of the target cell to NK cell recognition. Their expression appears to be regulated at different levels (epigenetic, transcriptional, post-transcriptional), but in this review we have summarized the current literature and highlighted the importance of the DDR in promoting NKG2D and DNAM-1 ligand expression, both at protein and mRNA levels, though the precise molecular mechanisms mediating these effects and the possible cooperation/regulation with upstream and downstream additional signaling pathways remain to be further clarified. However, DDR may represent a crucial point of convergence for ligand up-regulation, triggered by a big variety of circumstances and stressful stimuli.

## Conflict of Interest Statement

The authors declare that the research was conducted in the absence of any commercial or financial relationships that could be construed as a potential conflict of interest.
